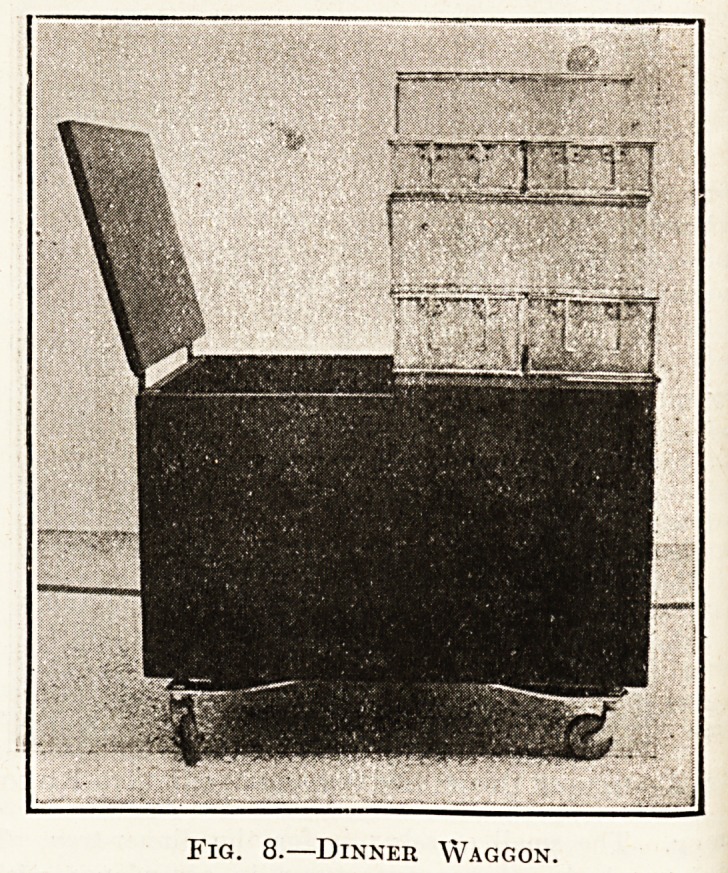# Some Home-Made Hospital Equipment

**Published:** 1914-10-10

**Authors:** 


### SOME HOME-MADE HOSPITAL EQUIPMENT.

### Examples from Leicester.

The following are examples of some of the new furniture and fittings used at the remodelled Children's Hospital of the Leicester Royal Infirmary. The whole of them have been designed and most of them made

Fig. 1.—Cot Bedstead.

by the infirmary's works department, and therefore possess a special interest.

Fig. 1 shows the cots. These are of the balcony type, easily moved by one person, 1¼-in. pillars, ⅝-in. filling diagonal springs bolted together, and rubber buffers. The stays for holding up the sides are of telescopic design running right across the cot, and very

Fig. 2.—Double Doors Converting Ward into Balcony.

strong. The small crossbar is for the dinner-tray. The cot is made by the manufacturer in accordance with a specification supplied to him, at a cost of 52s. 6d. each.

The double doors shown in fig. 2 have been inserted in place of the old windows in the wards. By this means the ward can be turned into a balcony at any time. The doors are made of teak 2½ in. thick, with draft-proof circular joints and hopper lights above.

Fig. 3.—Trolley for Washing Patients.

The approximate cost of double doors, frame and lights only, and no fixing, is £12.

Fig. 4a.—"Ambulance," showing Movable Shelves.

The trolley, used largely for washing the patients, is shown in fig. 3. This is made of aluminium throughout, and its approximate cost is £2.

Fig. 4b.—The "Ambulance," Side showing Cupboards and Drawers.

The ambulance shown in figs. 4a and 4b is a very useful piece of furniture, kept in the centre of the ward, fitted with all ward necessities for immediate use. It is made of teak with a glass top, and a special edge strip to prevent the moisture getting under the glass. It has shelves on one side all movable, and on the other cupboards and drawers. It is fitted with large Kendrick castors. The estimated cost is £15.

Fig. 5 shows the sterilising apparatus, one set of which, as shown, consisting of boiler, steriliser, and

Fig. 5.—Ward Sterilising Apparatus.

gas jet, is fitted in each ward. The stand is made of 1½-in. drawn-steel tube, 18 gauge, with special gun-metal joints, pegged and brazed together. The slab is of aluminium 3-16 in. thick. The steriliser and boiler are made of Muntz metal, 20-gauge, and white plated. The cost is about £10.

Fig. 6 shows an ice-chest. This is made with an inner and outer shell of teak with a cavity of ⅜ in.

Fig. 6.—Ice-chest.

filled with granulated cork. It is lined throughout with zinc. The upper portion is arranged for ice, the liquid running down to the floor through a tube; the lower portion is divided into two cupboards. The fittings are of mild steel copper plated and bronzed, and the estimated cost is £18.

Another trolley, shown in fig. 7, as the illustration indicates, is for the surgeon's use during an operation. It has a long arm for tucking under the table, and a tray to correspond coming over the table which can be raised or lowered. The cost is about £3.

The dinner waggon (fig. 8) is made of teak with flush

Fig. 7.—Trolley for Surgeon's Use.

panels and special hinges for allowing the lids to fall right back; it is fitted with springs bolted to the bottom and ball-bearing, rubber-tyred castors, two rigid and two movable. The food receptacles are of 18-gauge tin

Fig. 8.—Dinner Waggon.

with special drop handles. This will carry a dinner f[illegible] thirty patients. Several of these dinner waggons ha[illegible] been in use eight years. Their estimated cost is £[illegible] each.

## Figures and Tables

**Fig. 1. f1:**
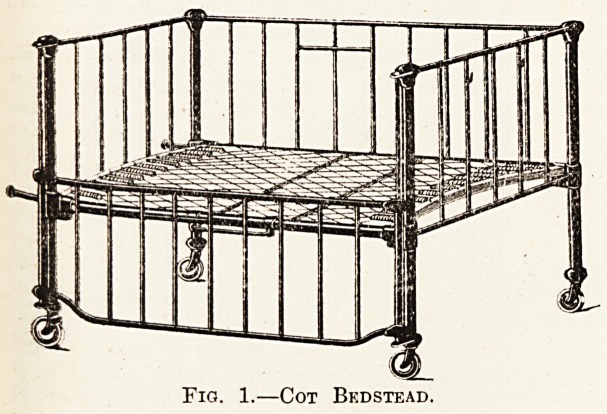


**Fig. 2. f2:**
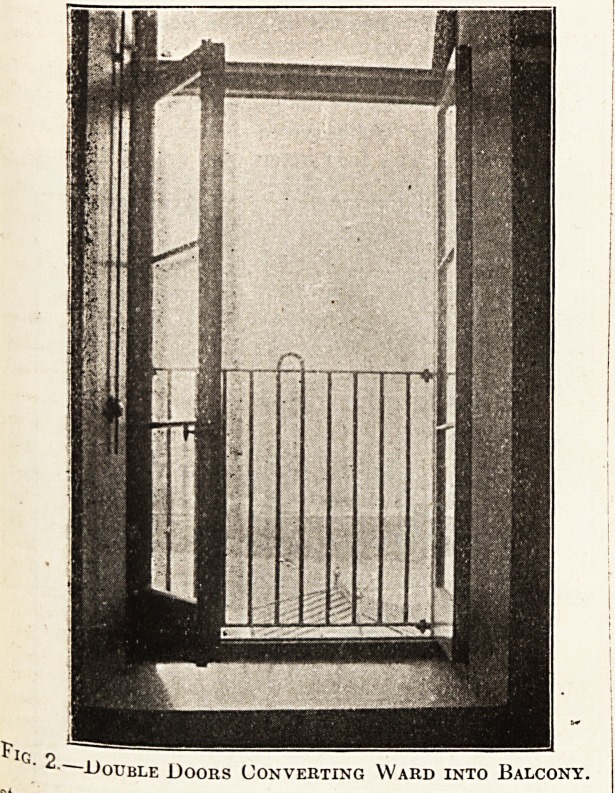


**Fig. 3. f3:**
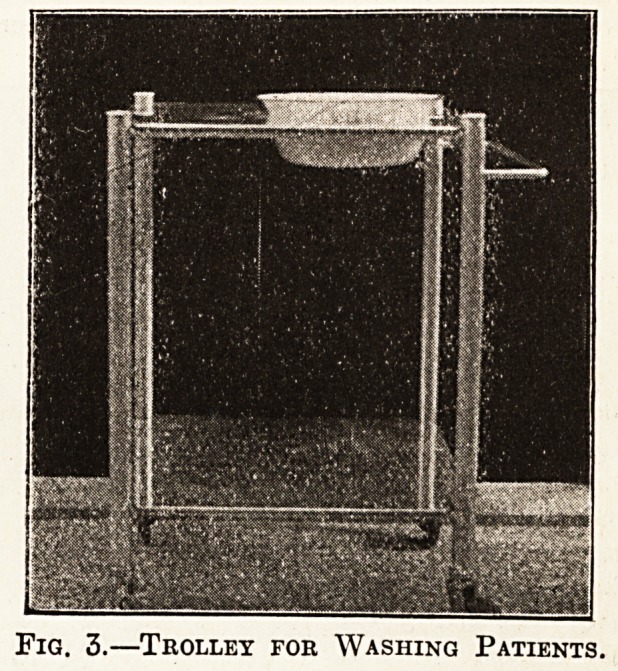


**Fig. 4a. f4:**
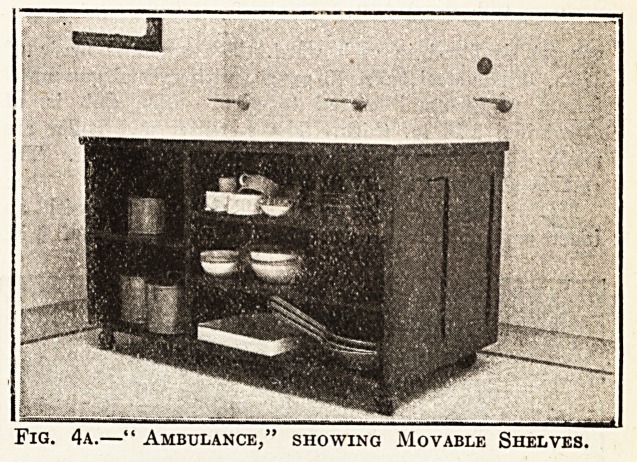


**Fig. 4b. f5:**
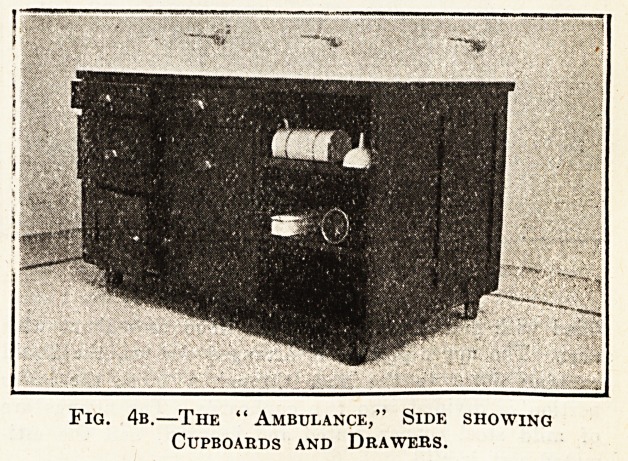


**Fig. 5. f6:**
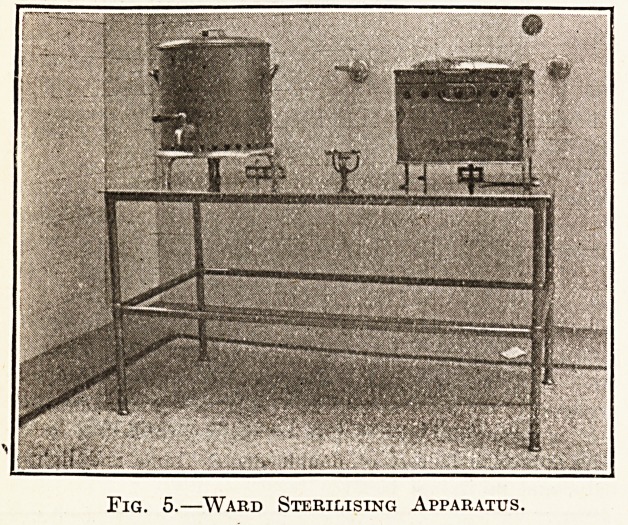


**Fig. 6. f7:**
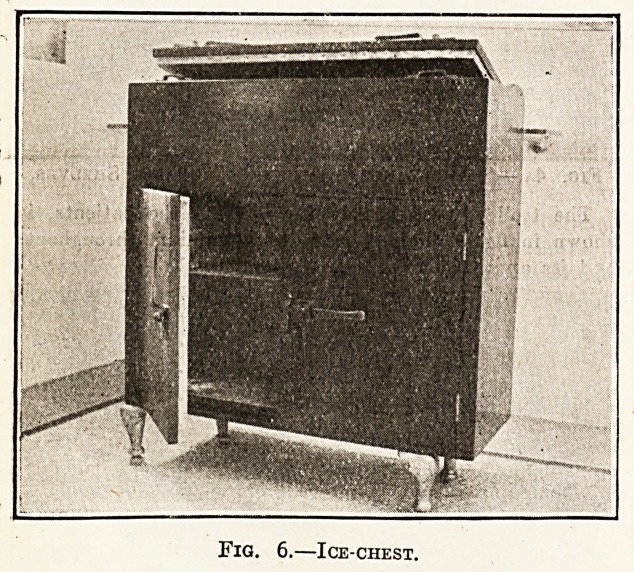


**Fig. 7. f8:**
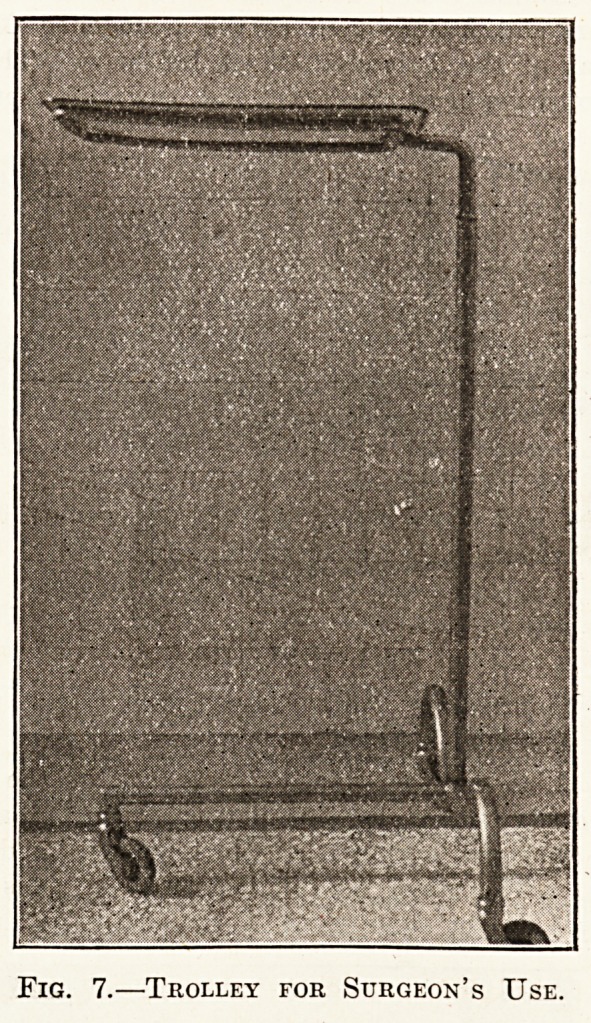


**Fig. 8. f9:**